# Breastfeeding, feeding practices and stunting in indigenous Ecuadorians under 2 years of age

**DOI:** 10.1186/s13006-022-00461-0

**Published:** 2022-03-05

**Authors:** Betzabé Tello, María F. Rivadeneira, Ana L. Moncayo, Janett Buitrón, Fabricio Astudillo, Andrea Estrella, Ana L. Torres

**Affiliations:** 1 Facultad de Medicina, Medicina Familiar, Pontificia Universidad Católica del Ecuador, Quito, Ecuador; 2 Facultad de Medicina Alberto Hurtado, Universidad Peruana Cayetano Heredia, Lima, Perú; 3 Facultad de Medicina, Instituto de Salud Pública, Pontificia Universidad Católica del Ecuador, 12 de Octubre and Roca, 1076 Quito, Ecuador; 4 Centro de Investigación para la Salud en América Latina (CISeAL), Facultad de Ciencias Exactas y Naturales, Pontificia Universidad Católica del Ecuador, Quito, Ecuador; 5 Facultad de Ciencias Exactas y Naturales, Matemática. Pontificia, Universidad Católica del Ecuador, Quito, Ecuador; 6 Facultad de Ciencias Humanas, Geografía. Pontificia, Universidad Católica del Ecuador, Quito, Ecuador; 7 Facultad de Enfermería, Nutrición. Pontificia, Universidad Católica del Ecuador, Quito, Ecuador

**Keywords:** Breastfeeding, Child feeding, Stunting, Complementary feeding, Indigenous population, Child public health

## Abstract

**Background:**

The indigenous child population in Ecuador has a high prevalence of stunting. There is limited evidence of the association between breastfeeding, feeding practices, and stunting in indigenous children. This study aimed to analyze the prevalence of breastfeeding and complementary feeding practices and explore their association with stunting in Ecuadorian indigenous children under two years of age.

**Methods:**

Cross-sectional study of secondary data analysis using the 2012 Ecuador National Health and Nutrition Study, in 625 children aged 0–23 months (48,069 expanded sample), representative for the indigenous population. Breastfeeding and complementary feeding indicators were analyzed by age groups. Timely initiation of breastfeeding (within one hour after birth), exclusive breastfeeding (infants under six months who received only breast milk for the previous day), and other indicators were measured. Chi-square test or Fisher's exact test and logistic regression for complex samples were used to explore association with demographic and socioeconomic factors and stunting.

**Results:**

Twenty-six-point eight percent of the children were stunted. Stunting occurred mainly in children with rural residence, on poor households, and where there were four or more children. Most of the children had a timely initiation of breastfeeding (69.5% for 0–12 months and 75.5% for 13–23 months) and exclusive breastfeeding up to six months (78.2%). Among children between 6–12 months of age, 99.3% continued to be breastfed. In children from ages 6 to 12 months, 32.5% received food with adequate dietary diversity. Lower percentages of complementary feeding occurred in the poorest, adolescent mothers or those with less education. Children who did not receive the minimum frequency of meals for their age had higher odds of stunting (OR 3.28; 95% CI 1.3, 8.27). Children from age 19 to 23 months who consumed foods rich in iron showed lower probabilities of stunting (OR 0.04; 95% CI 0.00, 0.51).

**Conclusions:**

Breastfeeding practices reached a prevalence of 70% or more, without being associated with stunting. Complementary feeding practices showed differences by socioeconomic condition. Not reaching the minimum meal frequency between 6 and 12 months of age was associated with stunting. Plans and strategies are necessary to promote adequate feeding and breastfeeding practices in the indigenous population.

**Supplementary Information:**

The online version contains supplementary material available at 10.1186/s13006-022-00461-0.

## Background

Childhood stunting is a public health problem that affects approximately 155 million children globally, which leads to higher child mortality, repeated infections, and fewer opportunities to play and learn [1, 2]. Among the most vulnerable groups affected by stunting are indigenous populations, which have historically suffered from health, economic and social inequity leading to less human development [1, 3]. In Latin America, the indigenous population represents 8.3% of the total inhabitants and, economically speaking, constitutes approximately 14% of the people classified as poor by income, and 17% of those classified as extremely poor by income [4].

In 2016, the rate of stunting in Latin America was 11%, 66% of which were in low- and middle-income countries, and 25% were in very low-income countries [5]. The risk of death is two times higher for an indigenous child than for a non-indigenous child [6]. In Ecuador, 7% of the population is indigenous and, as in most countries in Latin America, that population faces precarious living conditions. This phenomenon is reflected in the high prevalence of stunting within this population, 42.3% in the indigenous population compared to only 25.3% at the national level [7].

The growth and development of a child begins in its formation in the womb, and several factors will determine its nutritional status before and after birth; for instance, maternal health will influence in the weight at birth. Additionally, feeding practices such as breastfeeding and introduction and access to solid foods will impact on the child’s health. Other factors include exposure to contamination from non-human milk and other liquids, access (or lack thereof) to essential public services such as drinking water and health services, and socioeconomic and demographic status. These determinants could outweigh genetic predisposition in linear growth patterns and can lead to irreversible damage. These would trigger accumulated, permanent, and long-term effects on the growth and development of children, such as shorter height as adults, lower educational level, lower economic income, and decreased weight at birth [8–10].

Nutritional status and eating practices vary between populations and change over time; for instance, consumption decisions, lifestyle, and activities that have increasingly led to overweight and obesity problems in children and adolescents, are adding a double burden of malnutrition in children [3]. In this sense, it is essential to identify the situation of breastfeeding and feeding practices in the child population, mainly in the indigenous population, due to their greater vulnerability. This information would allow the generation of evidence to support the creation of policies and strategies to reduce the prevalence of malnutrition in indigenous children.

In Ecuador, there is limited epidemiological information on breastfeeding and feeding practices in indigenous populations and their relationship with stunting. The objective of this study is to identify the prevalence of breastfeeding and complementary feeding practices and explore their association with stunting in Ecuadorian indigenous children.

## Methods

A cross-sectional study of secondary data analysis was performed based on the National Health and Nutrition Survey (ENSANUT) carried out by the Ministry of Public Health and the National Institute of Statistics and Censuses (INEC) between 2011 to 2013 [7]. The survey was aimed to identify health and nutritional problems in the Ecuadorian population under 60 years of age. The sample was probabilistic, stratified, three-staged and by conglomerates, representative by age and ethnic group, including indigenous people [7]. In the first stage, the largest geographic-administrative divisions of the country (i.e., provinces) were classified into urban and rural areas, and a total of 64 census sectors were selected. In each census sector, 12 dwellings were randomly selected, a woman of childbearing age and a household member, of which were chosen by simple random sampling for each age group (a child under five years old, a teenager from 10 to 19 years old, and an adult from 20 to 59 years old). Nationally, 19,949 homes and 92,502 individuals were surveyed in total. Data collection was carried out through surveys containing information about household demographics, breastfeeding in children under three years old, health in children under five years old, women of childbearing age, anthropometry, and other factors related to the health of school children, teenagers and adults. The methodology, datasets and results of ENSANUT 2012 can be accessed at: https://www.ecuadorencifras.gob.ec/encuesta-nacional-de-salud-salud-reproductiva-y-nutricion-ensanut-2012/ [11].

For the present study, indigenous children from age 0 to 23 months with anthropometric data available from the ENSANUT 2012 database were included. Children who did not present anthropometric information or with information that could affect the validity of the data were excluded; this exclusion included children with physical problems, children who did not cooperate, those who refused to be measured, and cases with biologically implausible data. A total of 625 children aged 0–23 months of age, identified as indigenous by their mothers were included in this study. The expansion factor was applied to the sample, according to the methodology proposed in ENSANUT 2012 [7]. The calculated expanded sample was 48,069 indigenous children (95% CI 42,495–53,644).

Length measurement was carried out by previously trained personnel, using portable infantometers with a measuring range from 0 to 100 cm. Two length measurements were made, and a third if the difference between the previous two was ± 0.5 cm. The mean of the two measurements, or of the two closest, if there was a third measurement, was considered as the final value for length [7].

### Variables:

#### The variables analyzed in this study were:

Dependent variable:

- Stunting, defined by a Z-score of less than two standard deviations of length for age. Z values ​​were calculated using the WHO 2006 growth standard references [12].

Independent variables:

- Timely initiation of breastfeeding: child receives breastfeeding within the first hour of birth. This answers the question: At what time after birth did he / she begin to suckle or breastfeed?
- Exclusive breastfeeding (EBF) before six months: infants under six months who received only breast milk the day before. This corresponds to the question: Did you breastfeed your child during the day and the night? Complemented by the question: Did (the child) consume any liquid other than breast milk yesterday?
- Continued breastfeeding from age 6 to 23 months: in children from 6 to 23 months old, the mother continues to breastfeed. This corresponds to the question: Did you breastfeed your child every time he asked, that is, on demand since he was born? Did you give him breast milk yesterday, day and / or night?
- Consumption of foods other than breast milk: children who received some liquid or food other than breastfeeding during the previous day.
- Food diversity: children from 6 to 23 months old who received a number equal to or greater than four food groups the day before.
- Minimum frequency of meals for the respective age for children from 6 to 23 months old: minimum frequency of meals for their age (2 times for breastfed children from 6 to 8 months, 3 times for breastfed children from 9 to 23 months, 4 times for non-breastfed children 6 to 23 months).
- Consumption of foods rich in iron in children from 6 to 23 months old: children who, during the previous day, received a food rich in iron, or a food fortified with iron.
- For the variable timely initiation of breastfeeding, two age groups were differentiated: children ranging from age 0 to 12 months, and children ranging from age 13 to 23 months. EBF was only considered on children under 6 months old. The rest of indicators were analyzed withing groups from children of age 6 to 12 months, 13 to 18 months, and 19 to 23 months.

The following characteristics were considered as adjustment variables: a) related to the child: sex, presence of diarrhea and/or cough or runny nose the previous two weeks b) related to the child's mother: educational level (no studies, primary, high school, university), age, number of children the mother has had, marital status, height < 147 cm, c) related to the household: area of residence (urban, rural), economic quintile, household in extreme poverty (family income per capita less than USD $ 43.02 per month, as established by INEC for December 2012), Human Development Voucher (BDH; governmental monetary subsidy of $ 50 per month for families in extreme poverty).

### Statistical analysis

The association of demographic and socioeconomic factors with the indicators of lactation and complementary feeding was performed using the Chi-square test or Fisher's exact test. An analysis of the lactation and complementary feeding indicators was performed by age group according to each measure. In addition to the descriptive analysis, bivariable and multivariable analysis were performed, applying logistic regression for complex samples, adjusted for the following variables: sex, child’s age in months (where not analyzed for age groups), whether the child had diarrhea and/or cough or runny nose on the previous two weeks, area of residence, number of children, economic quintile, and mother's height. These were selected as adjusted variables mainly due to their statistically significant association with stunting. The variables *home in extreme poverty* and *receiving the Human Development Voucher* were excluded from the final model since these characteristics are already contained in the economic quintile. Odds Ratios (OR) and 95% Confidence Intervals (CI) were obtained as measure of association. A *p*-value < 0.05 was defined as significant. The statistical program SPSS® version 25 was used for the data analysis, and the macros for the WHO Stata® were used for the calculation of the anthropometric indicators, available at https://www.who.int/childgrowth/software/es/.

### Results

Of the 48,069 indigenous children under two years of age included in this study (*n* = 625), 27.88% were under six months, 30.33% between 6 and 12 months, 25.91% between 13 and 18 months, and 15.88% between 19 and 23 months of age. Additional file 1 shows the territorial location of the indigenous children included in this study (mainly in the Amazon region and central highlands). Characteristics of the sample are shown in Table 1.

**Table 1:** Characteristics of the sample of indigenous children under 2 years of age. National Health and Nutrition Survey, Ecuador, 2012 (*n* = 625, expanded = 48,069)

	**0–5 months**	**6–11 months & 29 days**	**12–23 months & 29 days**	**Total Sample**
**Variables**	***n*** **(%)**^b^	***n*** **(%)**^c^	***n*** **(%)**^d^	***n*** **(%)**^e^
**Children characteristics**				
Sex				
Male	5245 (39.1)	4212 (34.8)	10,688 (47.4)	20,145 (41.9)
Female	8158 (60.9)	7897 (65.2)	11,870 (52.6)	27,925 (58.1)
Diarrhea, cough, or runny nose on the last 2 weeks				
No	8114 (61.9)	6267 (52.8)	12,538 (56.7)	26,919 (57.2)
Yes	5004 (38.1)	5600 (47.2)	9563 (43.3)	20,167 (42.8)
**Mothers’ characteristics**				
Mother´s age (years)				
14–17	728 (5.4)	588 (4.9)	361 (1.6)	1678 (3.5)
18–25	6033 (45)	5174 (42.7)	10,091 (44.7)	21,297 (44.3)
26–35	5018 (37.4)	5309 (43.8)	8980 (39.8)	19,307 (40.2)
Over 35	1598 (11.9)	1039 (8.6)	3002 (13.3)	5639 (11.7)
Mother´s educational level^a^				
No studies	779 (5.9)	1085 (9.1)	1472 (6.7)	3336 (7.1)
Primary	7862 (59.9)	5953 (50.2)	13,706 (62)	27,520 (58.5)
High School	3471 (26.5)	3951 (33.3)	6040 (27.3)	13,462 (28.6)
University	1006 (7.7)	879 (7.4)	883 (4)	2768 (5.9)
Mother´s marital status^a^				
Civil union/married	10,710 (81.6)	9849 (83)	19,150 (86.6)	39,708 (84.3)
Single	1078 (8.2)	1160 (9.8)	1774 (8)	4012 (8.5)
Divorced/separated/widowed	1330 (10.1)	858 (7.2)	1178 (5.3)	3366 (7.2)
Number of children^a^				
1–3	8241 (62.8)	8666 (73)	15,731 (71.2)	32,639 (69.3)
4–7	3925 (29.9)	2533 (21.3)	4531 (20.5)	10,989 (23.3)
Over 7	952 (7.3)	668 (5.6)	1839 (8.3)	3459 (7.4)
Mother´s height < 147 cm				
> 147	8373 (62.6)	7952 (67)	11,809 (59.2)	28,134 (62.3)
< 147	5004 (37.4)	3923 (33)	8136 (40.8)	17,063 (37.7)
**Household characteristics**				
Area of residence				
Urban	4077 (30.5)	2933 (24.2)	6671 (29.7)	13,681 (28.6)
Rural	9300 (69.5)	9176 (75.8)	15,763 (70.3)	34,239 (71.5)
Economic quintile				
5 (richest)	25 (0.2)	84 (0.7)	377 (1.7)	487 (1)
4	1014 (7.6)	758 (6.3)	276 (1.2)	2048 (4.3)
3	264 (2)	1686 (13.9)	3204 (14.2)	5154 (10.7)
2	3921 (29.3)	2616 (21.6)	3415 (15.1)	9952 (20.7)
1 (poorest)	8180 (61)	6965 (57.5)	15,285 (67.8)	30,430 (63.3)
Home in extreme poverty				
No	7360 (54.9)	7823 (64.6)	12,645 (56.1)	27,828 (57.9)
Yes	6043 (45.1)	4287 (35.4)	9912 (43.9)	20,241 (42.1)
Human Development Voucher				
No	8248 (61.7)	7047 (58.2)	11,392 (50.8)	26,687 (55.7)
Yes	5129 (38.3)	5063 (41.8)	11,042 (49.2)	21,233 (44.3)
**Dependent variable**				
Stunting				
Yes	1270 (9.5)	1540 (12.7)	10,065 (44.6)	12,875 (26.8)
No	12,133 (90.5)	10,570 (87.3)	12,492 (55.4)	35,195 (73.2)

^a^missing data for 9 children

^b^Sample *n* = 166, expanded = 13,403 (95% CI 10,176, 16,630); ^c^Sample *n* = 170, expanded = 12,110 (95% CI 9296, 14,923); ^d^Sample *n* = 289, expanded = 22,557 (95% CI 17,421, 27,693); ^e^Sample *n* = 625, expanded = 48,069 (95% CI 42,495, 53,644)

Table 2 shows the prevalence of breastfeeding indicators in indigenous children. The 69.5% (95% CI 61.2%, 76.7%) of indigenous children between 0 and 12 months of age and 75.5% (95% CI 64.4%, 83.9%) of children ranging from age 13 to 23 months of age were breastfed in the first hour after birth. The children of ages 0 to 12 months from mothers without education (75.6%; 95% CI 50.5%, 90.4%), living in rural areas (75.9%; 95% CI 69.6%, 81.3%) and from households in extreme poverty (78.6%; 95% CI 70.3%, 85%) had significantly higher prevalence of breastfeeding in the first hour than the children of women with a university education, living in urban areas and without extreme poverty (20.9%; CI 95% 5.7%, 53.7%; 20.9%; CI 95% 5.7%, 53.7%; 63.5%; CI 95% 51.6%, 74%, respectively).

**Table 2:** Prevalence of breastfeeding and feeding indicators, indigenous children. Ecuador, 2012. [% (95% CI) = percentage (95% confidence interval expanded sample)]

	**Timely initiation of breastfeeding**^**a**^** (0, 12 months)**	**Timely initiation of breastfeeding**^**a**^** (13, 23 months)**	**Exclusive breastfeeding**^**b**^** in children under 6 months**	**Continued breastfeeding**	**Continued breastfeeding**	**Continued breastfeeding**	**Received food the day before**	**Received food the day before**	**Received food the day before**
				**(6, 12 months)**	**(13, 18 months)**	**(19, 23 months)**	**(6, 12 months)**	**(13, 18 months)**	**(19, 23 months)**
	**% (95% CI) †**	**% (95% CI) ††**	**% (95% CI) †††**	**% (95% CI) ††††**	**% (95% CI) †††††**	**% (95% CI) ††††††**	**% (95% CI) ††††**	**% (95% CI) †††††**	**% (95% CI) ††††††**
**Meets indicator**	69.5 (61.2, 76.7)	75.5 (64.6, 83.9)	78.2 (68, 85.8)	99.3 (97.7, 99.8)	98.5 (96.1, 99.4)	94.3 (85.3, 98)	72.5 (61.7, 81.2)	98.8 (95.4, 99.7)	99.1 (93.4, 99.9)
**Children characteristics**									
Child´s gender									
Male	73.9 (61.6, 83.3)	77.4 (64.9, 86.3)	83.5 (70.4, 91.4)	99.5 (96.3, 99.9)	97.5 (92.9, 99.2)	90.8 (73.6, 97.2)	72.8 (60.1, 82.6)	97.7 (91.4, 99.4)	100 (…)
Female	66.8 (55.3, 76.6)	73.8 (56.5, 85.9)	74.8 (60.3, 85.2)	99.2 (96.5, 99.8)	99.6 (96.8, 99.9)	96.7 (79.2, 99.6)	72.3 (56, 84.3)	100 (…)	98.5 (89.1, 99.8)
Diarrhea, cough, or runny nose on the last 2 weeks									
No	67 (55.9, 76.4)	74.6 (57.8, 86.3)	86.1 (74.7, 92.9)	100 (…)	98.9 (94.9, 99.8)	91.3 (73, 97.6)	71.2 (52.6, 84.6)	100 (…)	100 (…)
Yes	72 (57.4, 83.1)	75.8 (64.2, 84.6)	66.9 (46.1, 82.7)	98.5 (95.2, 99.6)	97.8 (93.1, 99.3)	97.3 (91.1, 99.2)	75.5 (63.6, 84.5)	96.9 (88.2, 99.2)	98 (86.3, 99.7)
**Mother’s characteristics**									
Mother´s age (years)									
14–17	89.5 (70.7, 96.8)^c^	60.6 (19, 90.9)	86.6 (62.2, 96.2)	100 (…)	88.7 (44.5, 98.7)	100 (…)	61.1 (25.6, 87.8)	100 (…)	100 (…)
18–25	55.4 (42.1, 67.9)^**^	74 (54.9, 87)	80.2 (63.2, 90.6)	99.3 (95, 99.9)	99.2 (96.5, 99.8)	93.9 (66.5, 99.2)	79.8 (67.9, 88.1)	97.6 (90.8, 99.4)	100 (…)
26–35	81.9 (71.8, 88.9)	80.6 (65.3, 90.2)	79.5 (61, 90.6)	99.1 (96.1, 99.8)	98.7 (90.5, 99.8)	93.8 (78.8, 98.4)	65.8 (45.6, 81.5)	100 (…)	98.3 (87.8, 99.8)
Over 35	75.7 (60, 86.6)	67.6 (46.9, 83.1)	61.9 (36.2, 82.3)	100 (…)	97.2 (81, 99.6)	96.8 (78.7, 99.6)	71 (45.9, 87.6)	100 (…)	100 (…)
Mother´s educational level									
No studies	75.6 (50.5, 90.4)^c^	90.1 (49.6, 98.8)	91.8 (66.5, 98.4)	100 (…)	100 (…)	40.8 (4.8, 90.3)^d^	69.6 (31.9, 91.8)	100 (…)	100 (…)
Primary	81.4 (74.1, 87)	71.3 (55.3, 83.3)	76.3 (61.5, 86.6)	99 (95.6, 99.8)	99.1 (95.9, 99.8)	95.4 (82.9, 98.9)	70.6 (52.9, 83.7)	98.5 (92.4, 99.7)	98.5 (89.4, 99.8)
High School	57.8 (44.1, 70.4)	81 (70.5, 88.4)	76.9 (59.1, 88.4)	99.4 (96, 99.9)	96 (86.6, 98.9)	98.5 (89.7, 99.8)	73.8 (59.6, 84.3)	99 (92.9, 99.9)	100 (…)
University	20.9 (5.7, 53.7)^**^	69.4 (32.3, 91.5)	95.1 (67.3, 99.5)	100 (…)	100 (…)	100 (…)	93.3 (56.8, 99.3)	100 (…)	100 (…)
Mother´s marital status									
Civil union/married	67.1 (57.7, 75.3)	74.8 (62.8, 83.9)^c^	82.4 (72.3, 89.3)	99.1 (97.1, 99.7)	98.2 (95.2, 99.3)^c^	94.1 (84.7, 97.9)^c^	72.4 (59.7, 82.3)	98.6 (94.4, 99.6)	99 (93.1, 99.9)
Single	78.4 (50.3, 92.8)	54.1 (27.7, 78.5)	77.5 (44.9, 93.6)	100 (…)	100 (…)^*^	100 (…)^*^	81 (59.1, 92.7)	100 (…)	100 (…)
Divorced/widowed	78.1 (54.7, 91.4)	100 (…)^**^	50.9 (21.6, 79.6)	100 (…)	100 (…)^*^	-	68.9 (29.9, 92)	100 (…)	-
Number of children									
1, 3	64.2 (53, 74.1)^c^	74.6 (60.1, 85.2)	81.3 (68.5, 89.7)	99.5 (97.9, 99.9)	98.6 (95.9, 99.5)	96.2 (81.9, 99.3)	73.8 (59.8, 84.2)	98.4 (93.6, 99.6)	98.5 (89.7, 99.8)
4, 7	79.6 (70, 86.7)^*^	78.3 (64.5, 87.7)	74.5 (52.9, 88.4)	98.4 (88.9, 99.8)	97.3 (82.5, 99.6)	91.3 (64, 98.4)	73.8 (56.1, 86.2)	100 (…)	100 (…)
Over 7	82.8 (58.3, 94.3)	71.1 (45.1, 88.1)	74.2 (39.8, 92.6)	100 (…)	100 (…)	86.8 (54.1, 97.3)	63.3 (27.8, 88.5)	100 (…)	100 (…)
**Household characteristics**									
Area of residence									
Urban	52.3 (32.3, 71.6)^c^	74.8 (47, 90.9)	76.4 (51.5, 90.8)	100 (…)	98.7 (89.9, 99.8)	98.1 (84.5, 99.8)	74.8 (36.4, 93.9)	97.9 (85.2, 99.7)	100 (…)
Rural	75.9 (69.6, 81.3)^*^	76.1 (65, 84.5)	78.9 (67.9, 86.8)	99.1 (97, 99.7)	98.4 (95.5, 99.4)	93.2 (81.7, 97.7)	71.8 (62.6, 79.5)	99.3 (97.3, 99.8)	98.8 (91.5, 99.8)
Economic quintile									
5 (richest)	89.5 (44.2, 98.9)	72.3 (17.9, 96.9)	100 (…)^**^	100 (…)	100 (…)	100 (…)	100 (…)^**^	100 (…)	100 (…)
4	32.2 (7.3, 74.1)^*^	76 (21.5, 97.3)	100 (…)^**^	96.5 (68.3, 99.7)	70 (12.9, 97.4)	100 (…)	100 (…)^**^	100 (…)	100 (…)
3	85.3 (61.6, 95.5)	73.7 (36.9, 93.1)	72.4 (25.7, 95.2)	98.6 (89.5, 99.8)	100 (…)	95.4 (68.2, 99.5)	53.9 (18.7, 85.6)	100 (…)	100 (…)
2	63.4 (42.8, 80)	60.1 (26.2, 86.5)	77.2 (51.6, 91.5)	100 (…)	98.8 (91.2, 99.9)	100 (…)	70.4 (50.3, 84.8)	100 (…)	100 (…)
1 (poorest)	73.7 (66.2, 80)^c^	79.2 (69.8, 86.3)	76 (64.1, 85)^c^	99.4 (96, 99.9)	98.7 (95.7, 99.6)	93.6 (82.4, 97.8)	74.5 (63.7, 82.9)^c^	98.1 (92.7, 99.5)	98.9 (91.9, 99.9)
Home in extreme poverty									
No	63.5 (51.6, 74)^*^	71.5 (55.1, 83.7)	80.1 (65.8, 89.4)	99.2 (96.7, 99.8)	97.7 (93.4, 99.2)	97.5 (92.3, 99.3)	75.2 (59.5, 86.3)	99.6 (97.1, 99.9)	100 (…)
Yes	78.6 (70.3, 85)^c^	80.3 (67.5, 88.9)	75.8 (61, 86.3)	99.4 (96, 99.9)	99.5 (96.5, 99.9)	90.8 (71.8, 97.4)	67.5 (53.2, 79.1)	97.8 (89.6, 99.6)	98.1 (86.3, 99.8)
Human Development Voucher									
No	65.1 (52.4, 76)	79.7 (60.8, 90.8)	76.6 (61.8, 86.9)	99.7 (97.7, 100)	98.4 (94.3, 99.6)	98.7 (91.2, 99.8)	71.8 (55, 84.1)	99.2 (96.8, 99.8)	97.4 (82.7, 99.6)
Yes	76.3 (67.9, 83.1)	72 (59.4, 81.8)	80.6 (67.8, 89.1)	98.7 (94.5, 99.7)	98.6 (94.3, 99.7)	91.9 (78.1, 97.3)	73.7 (59.2, 84.4)	98.3 (88.9, 99.8)	100 (…)

^†^Sample *n* = 369, expanded = 27,983 (95% CI 23,581, 32,385); ††Sample *n* = 256, expanded = 120,087 (95% CI 15,415, 24,759); ††† Sample *n* = 166, expanded = 13,403 (95% (CI 10,176, 16,630); ††††Sample *n* = 203, expanded = 14,580 (95% CI 11,656, 17,505); †††††Sample *n* = 153, expanded = 12,453 (95% CI 8672, 16,234); †††††† Sample *n* = 103, expanded = 7634 (95% CI 5358, 9909)

^a^Timely initiation of breastfeeding = breastfeeding within one hour after birth

^b^Exclusive breastfeeding (EBF) = infants under 6 months who received only breast milk for the previous 24 h

^c^Reference category

^d^Few observations may cause under or over estimation

Statistically significant difference, *p* value < 0.05. ** Statistically significant difference, *p* value < 0.01

The 78.2% (95% CI 64.6%, 83.9%) of children under six months received exclusive breastfeeding. Women with the best economic quintile maintained EBF in a significantly higher percentage than women from the poorest quintile. (100% vs. 76%; 95% CI 64.1%, 85%). Continuous breastfeeding was maintained in more than 90% of the children of all age groups analyzed.

Regarding receiving food the day before, the prevalence was lower in those 6–12 months (72.5%; 95% CI 61.7%, 81.2%), compared with those 13–18 months (98.8%; 95% CI 95.4%, 99.7%) and 19–23 months (99.1%; 95% CI 93.4%, 99.9%). Children from ages 6 to 12 months from the poorest quintile presented a significantly lower prevalence of having received food the day before, (74.5%; 95% CI 63.7%, 82.9%) when compared to those of the richest quintile (100%) (Table 2).

Table 3 shows the prevalence of complementary feeding indicators in indigenous children. The 32.5% (95% CI 23.6%, 42.8%) of children from 6 to 12 months, 55.6% (95% CI 42%, 68.4%) of the group from 13 to 18 months, and 63.3% (95% CI 47.8%, 76.5%) of the group from 19 to 23 of children had a diverse diet, that is, they received at least four food groups the previous day. Children aged 13 to 18 months who resided in the rural area (66.4%; 95% CI 56.7%, 74.8%) and from the economic quintile 4 (100%) presented better percentages of a diverse diet, compared to those that lived in the urban area (36.7%; 95% CI 14.3%, 66.8%) and from the poorest quintile 1 (54%; 95% CI 41.4%, 66.1%). The 100% of children aged 19–23 months in the highest economic quintile received a diverse diet, compared to only 57.1% (95% CI 39.3%, 73.2%) in the poorest quintile.

**Table 3:** Prevalence of complementary feeding indicators in indigenous children from 6 to 23 months, according to socio, economic characteristics Ecuador, 2012. [% (95% CI) = percentage (95% confidence interval expanded sample)]

	**Food diversity**	**Food diversity**	**Food diversity**	**Minimum meal frequency**	**Minimum meal frequency**	**Minimum meal frequency**	**Consumption of foods rich in iron**	**Consumption of foods rich in iron**	**Consumption of foods rich in iron**
	**(6, 12 months)**	**(13, 18 months)**	**(19, 23 months)**	**(6, 12 months)**	**(13, 18 months)**	**(19, 23 months)**	**(6, 12 months)**	**(13, 18 months)**	**(19, 23 months)**
	**% (95% CI)†**	**% (95% CI)††**	**% (95% CI)†††**	**% (95% CI)†**	**% (95% CI)††**	**% (95% CI)†††**	**% (95% CI)†**	**% (95% CI)††**	**% 95% CI)†††**
**Meets indicator**	32.5 (23.6, 42.8)	55.6 (42, 68.4)	63.3 (47.8, 76.5)	78.8 (70.5, 85.2)	58 (43.8, 71)	67.8 (50.2, 81.5)	49.6 (39.4, 59.8)	65 (48, 78.8)	77.7 (66, 86.2)
**Children characteristics**									
Child´s gender									
Male	26.9 (15.8, 41.9)	56.2 (37.3, 73.5)	64.8 (44.4, 80.9)	79.3 (67.9, 87.4)	63.9 (44.6, 79.5)	81.9 (66.1, 91.3)	46.4 (33.5, 59.8)	63.6 (41.8, 81)	81.9 (65.9, 91.4)
Female	35.8 (23.8, 49.8)	54.9 (35.6, 72.9)	62.3 (41.6, 79.4)	78.5 (67, 86.7)	51.7 (32.4, 70.5)	58.3 (34.5, 78.9)	51.5 (37.3, 65.4)	66.5 (40.4, 85.3)	74.9 (57.5, 86.8)
Diarrhea, cough, or runny nose on the last 2 weeks									
No	35.6 (22.4, 51.5)	55.2 (36, 72.9)	60.3 (39.8, 77.6)	79.7 (67.1, 88.3)	57.5 (37.8, 75.1)	71.4 (52.4, 85)	53 (36.7, 68.7)	66.4 (40.9, 85)	84.4 (67.8, 93.3)
Yes	30.2 (19.8, 43.2)	55.1 (38.4, 70.8)	67.3 (45, 83.8)	77.1 (66.2, 85.3)	57.6 (38, 75.1)	65.9 (34.7, 87.6)	47.7 (35.6, 60.1)	61.7 (41.4, 78.6)	72.5 (52.1, 86.4)
**Mother’s characteristics**									
Mother´s age (years)									
14–17	17 (3.9, 50.4)	100 (…)	22 (1.6, 83.3)	82.2 (52.7, 95.1)	78.7 (26, 97.5)	100 (…)	18.7 (4.9, 51)^a^	100 (…)^**^	78 (16.7, 98.4)
18–25	34.8 (22, 50.3)	97.6 (90.8, 99.4)	49.8 (30, 69.6)	77.2 (65.2, 86)	54.4 (31.9, 75.2)	68.1 (47, 83.6)	55.9 (41.3, 69.6)^**^	51.9 (30.8, 72.4)^a^	74.1 (52.6, 88)
26- 35	33.8 (19.8, 51.4)	100 (…)	70.6 (49, 85.7)	79.8 (65.8, 89.1)	58 (35.8, 77.4)	65.5 (36.2, 86.3)	44.4 (28.6, 61.4)	71.6 (44.9, 88.6)	80.4 (61.8, 91.2)
Over 35	20.6 (5.9, 51.8)	100 (…)	68.6 (39.3, 88)	80.5 (49.1, 94.6)	66.5 (39.5, 85.8)	71.5 (40.4, 90.3)	54.8 (33.7, 74.3)^*^	88.8 (72, 96)^**^	74.2 (43, 91.6)
Mother´s educational level									
No studies	13.4 (1.7, 57.5)	100 (…)^a^	,	73.3 (33.2, 93.8)	34.9 (6.7, 79.9)	40.8 (4.8, 90.3)	69.6 (31.9, 91.8)	47.4 (9, 89.1)	100 (…)^**^
Primary	30.6 (18.9, 45.6)	44 (26.8, 62.7)^**^	68.2 (47.4, 83.7)	77.9 (64.8, 87.1)	56 (36, 74.3)	65.4 (39.8, 84.4)	43 (29.7, 57.4)	58.4 (37.6, 76.6)	77.7 (60.7, 88.7)^a^
High School	41.6 (28, 56.6)	60 (39.9, 77.2)	61.2 (38.9, 79.6)	77.7 (66, 86.3)	66.6 (44.8, 83)	79.7 (62.1, 90.4)	50.5 (36.4, 64.6)	83.9 (71.3, 91.6)	75.9 (55.1, 89)
University	28.4 (5.3, 73.8)	95.4 (69.6, 99.5)	84.6 (35.6, 98.2)	90.9 (56.7, 98.7)	82.7 (42.8, 96.8)	66.4 (22.3, 93.1)	81.5 (41.3, 96.5)	93.6 (70.3, 98.9)	100 (…)^**^
Mother´s marital status									
Civil union/married	32.2 (22.5, 43.7)	51.2 (37, 65.2)	64 (47.9, 77.4)	78 (68.6, 85.1)	61.9 (46.1, 75.5)^a^	68.6 (50, 82.7)	49 (37.4, 60.7)	64.1 (45.2, 79.5)^a^	79.2 (67.6, 87.5)
Single	43.2 (17.7, 72.9)	75.5 (48.5, 91)	45.8 (6.8, 90.8)	87.4 (60.8, 96.9)	58.4 (29.6, 82.4)	76.3 (20.5, 97.6)	65.2 (42.2, 82.7)	90.4 (70.5, 97.4)^*^	45.8 (6.8, 90.8)
Divorced/widowed	23.3 (5.3, 62.2)	72 (28, 94.4)	-	66.6 (27.9, 91.2)	18.7 (3.1, 62.3)^*^	-	39.9 (12.8, 75.1)	45.8 (8.9, 87.9)	-
Number of children									
1, 3	33.6 (23, 46.2)	53.6 (36.1, 70.2)	64.4 (44.3, 80.4)	79.9 (70.6, 86.8)	54 (36.3, 70.7)	68.9 (42.2, 87)	52.6 (39.9, 64.9)	58.9 (38.7, 76.5)^a^	80.7 (63.4, 91)
4, 7	32.4 (17.8, 51.5)	57.5 (42.2, 71.5)	57.3 (35.7, 76.4)	72.9 (53, 86.6)	60.1 (44.4, 74)	62.3 (40.8, 79.8)	49.1 (32.4, 66.1)	75.9 (62.2, 85.8)	71.8 (51.1, 86.1)
Over 7	26.2 (5.4, 69)	61.9 (31.1, 85.4)	83.1 (51.6, 95.8)	78.6 (30.7, 96.8)	78 (47.1, 93.4)	94 (63.4, 99.3)	23 (4.6, 64.8)	85.4 (63.4, 95.2)^*^	87.6 (55.8, 97.5)
**Household characteristics**									
Area of residence									
Urban	30.8 (12.9, 57.2)	36.7 (14.3, 66.8)^a^	82.2 (50, 95.5)	76.5 (54.2, 90)	39.5 (15.4, 70.2)	41 (12.2, 77.7)	55.5 (27.1, 80.6)	31 (13.5, 56.4)^a^	92.7 (76.9, 98)^a^
Rural	33 (23.6, 44)	66.4 (56.7, 74.8)^*^	57.6 (40.9, 72.7)	79.5 (70.5, 86.3)	67.7 (56.4, 77.2)	75.4 (61.7, 85.3)	47.7 (38, 57.5)	83.1 (72.9, 90.1)^**^	73.2 (59.7, 83.4)^*^
Economic quintile									
5 (richest)	74.4 (18.2, 97.4)	55.6 (6.9, 95.5)	100 (…)^**^	100 (…)^**^	55.6 (6.9, 95.5)	-	100 (…)^**^	100 (…)^**^	100 (…)^**^
4	6.1 (0.7, 36.5)^b^	100 (…)^**^	100 (…)^**^	87.6 (45.9, 98.3)	70 (12.9, 97.4)	100 (…)^**^	93.4 (61.6, 99.2)^**^	95 (60.3, 99.6)^*^	100 (…)^**^
3	34.1 (11.8, 66.8)	60.3 (16, 92.4)	93 (68.4, 98.8)^**^	82.6 (53.3, 95.2)	54.6 (14.8, 89.3)	91.2 (62.7, 98.5)^*^	49.7 (17.5, 82.1)	37.2 (9.6, 76.7)	93 (69.2, 98.8)^*^
2	45 (25.8, 65.9)	53 (19.9, 83.6)	85.5 (50.3, 97.2)^*^	80.1 (63.1, 90.5)	40.7 (15.1, 72.5)	59 (20.5, 89)	40.1 (21.3, 62.2)	60.6 (21.8, 89.5)	95 (68, 99.4)^*^
1 (poorest)	28.9 (19.4, 40.8)^a^	54 (41.4, 66.1)^a^	57.1 (39.3, 73.2)^a^	76 (65.5, 84.1)^a^	64.3 (49.3, 77)	66.9 (46.4, 82.6)^a^	47.6 (36.5, 59.1)^a^	73 (56, 85.1)^a^	73.9 (60, 84.2)^a^
Home in extreme poverty									
No	36.1 (24.4, 49.8)	58.6 (38.8, 75.9)	74.8 (56.4, 87.2)	79.8 (69.4, 87.3)	59 (40.2, 75.4)	81.4 (67.4, 90.3)^*^	51.5 (37.7, 65)	69.8 (44.4, 87)	82.9 (71.1, 90.6)
Yes	25.6 (15, 40.2)	51.8 (32.3, 70.7)	51 (27, 74.5)	76.9 (62.6, 86.8)	56.8 (35.4, 75.9)	52.7 (28.9, 75.3)^a^	46.1 (33, 59.7)	58.7 (37, 77.5)	72 (50.9, 86.5)
Human Development Voucher									
No	34.4 (22.3, 49)	52.8 (32, 72.8)	58.2 (37.8, 76.1)	83.1 (73.2, 89.9)	48.9 (29.3, 68.9)	78.3 (57.6, 90.5)	48.9 (34.2, 63.7)	55.7 (32.5, 76.7)	71 (49.7, 85.8)
Yes	29.4 (18.3, 43.7)	59.9 (46.8, 71.7)	65.6 (44.9, 81.7)	72 (58.2, 82.7)	69.1 (56.4, 79.5)	61.8 (38.7, 80.5)	50.7 (36.3, 65)	76.4 (63.9, 85.5)	81 (67, 89.9)

^†^Sample *n* = 203, expanded = 14,580 (95% CI 11,656, 17,505); ††Sample *n* = 153, expanded = 12,453 (95% CI 8672, 16,234); ††† Sample *n* = 103, expanded = 7634 (95% CI 5358, 9909)

^*^Statistically significant difference, *p* value < 0.05. ** Statistically significant difference, *p* value < 0.01

^a^Reference Category

^b^Few observations may cause under or overestimation

The 78.8% (95% CI 70.5%, 85.2%), 58% (95% CI 43.8%, 71%), and 67.8% (95% CI 50.2%, 81.5%) of children from the 6 to12 months, 13 to 18 months and 19 to 23 months, respectively, received the minimum frequency of meals for their age. Children in the 6 to 12 months age group from the highest economic quintile met 100% minimum meal frequency, significantly higher than those from the poorest quintile (76%, 95% CI 65.5%, 84.1%). In the 13–18 month group, children of widowed or divorced mothers had a significantly lower prevalence of minimum meal frequency, compared to children of married mothers (18.7%, 95% CI 3.1%, 62.3% vs. 61.9%, 95% CI 46.1%, 75.5%, respectively). Only 52.7% (95% CI 28.9%, 75.3%) of children aged 19 to 23 months in extreme poverty received the minimum frequency of meals.

The 49.6% (95% CI 39.4%, 59.8%), 65% (95% CI 48%, 78.8%), and 77.7% (95% CI 66%, 86.2%) of children from the 6 to12 months, 13 to 18 months and 19 to 23 months, respectively consumed foods rich in iron the day before, respectively. Children in the 6 to 12 month age group, whose mothers were adolescents and belonged to the poorest quintile received significantly less iron-rich foods (18.7%, 95% CI 4.9%, 51%; 47.6%, 95% CI 36.5%, 59.1%, respectively), compared to those children whose mothers aged over 35 years and belonged to the richest economic quintile (54.8%, 95% CI 33.7%, 74.3%; 100%, respectively). In the 19 to 23 month age group, children whose mothers had university education showed a higher proportion of being fed foods rich in iron (100%) when compared to mothers with only primary schooling (77.7%, 95% CI 60.7%, 88.7%), and higher for those children living in the urban area (92.7%, 95% CI 76.9%, 98%) when compared to those living in the rural area (73.2%, 95% CI 59.7%, 83.4%), and from the richest economic quintile (100%) when compared to those in the poorest quintile (73.9%, 95% CI 60%, 84.2%) (Table 3).

The 26.8% of the children presented stunting. Children from age 13 to 18 months and 19 to 23 months had higher probability of being stunted (OR 5.03, 95% CI 2.26, 11.21, and OR 21.99, 95% CI 8.37, 57.77) compared to those under six months (Table 4).

**Table 4:** Association between stunting (length, for, age Z, score < 2 SD) and characteristics of the children. mother and household. National Health and Nutrition Survey. Ecuador. 2012 (*n* = 625, expanded sample = 48,069)

**Variables**	Stuntig	Stuntig	OR (95% CI)
	*n* (expanded sample)	Expanded simple	
		% (95% CI)	
**Children characteristics**			
Sex			
Male	96 (6503)	50.5 (38.8, 62.1)	1
Female	79 (6371)	49.5 (37.9, 61.2)	0.62 (0.34, 1.12)
Age			
0–5 months	16 (1270)	9.9 (5.5, 17.1)	1
6–12 months	34 (1989)	15.4 (10, 23.1)	1.51 (0.7, 3.27)
13–18 months	62 (4295)	33.4 (24.5, 43.6)	5.03 (2.26, 11.21)^**^
19–23 months	63 (5322)	41.3 (32.8, 50.4)	21.99 (8.37, 57.77)^**^
Diarrhea, cough, or runny nose on the last 2 weeks			
No	84 (7187)	57 (47.8, 65.8)	1
Yes	89 (5414)	43 (34.2, 52.2)	1.01 (0.64, 1.59)
**Mother’s characteristics**			
Mother´s age (years)			
14–17	8 (480)	3.7 (1.6, 8.5)	1
18–25	75 (4914)	38.2 (30.3, 46.7)	0.75 (0.27, 2.07)
26–35	61 (5149)	40 (31.6, 49)	0.91 (0.33, 2.48)
Over 35	31 (2332)	18.1 (12.4, 25.7)	1.76 (0.57, 5.46)
Mother´s educational level			
No studies	6 (605)	4.8 (2, 11.3)	1
Primary	99 (8155)	64.7 (56.2, 72.3)	1.9 (0.61, 5.96)
High School	62 (3530)	28 (21.2, 36)	1.6 (0.49, 5.23)
University	6 (312)	2.5 (0.9, 6.5)	0.57 (0.13, 2.55)
Mother´s marital status			
Civil union/married	157 (11,779)	93.5 (87.9, 96.6)	1
Single	8 (387)	3.1 (1.4, 6.5)	0.25 (0.1, 0.63)^**^
Divorced/separated/widowed	8 (435)	3.5 (1.3, 8.9)	0.35 (0.11, 1.12)
Number of children			
1–3	96 (7106)	56.4 (47.5, 64.9)	1
4–7	55 (3857)	30.6 (22.1, 40.7)	1.94 (1.13, 3.33)^*^
Over 7	22 (1639)	13 (8.1, 20.2)	3.24 (1.7, 6.15)^**^
Mother´s height < 147 cm			
> 147 cm	103 (6554)	59.7 (47.6, 70.7)	1
< 147 cm	50 (4426)	40.3 (29.3, 52.4)	1.15 (0.61, 2.16)
**Household characteristics**			
Area of residence			
Urban	38 (2490)	19.3 (13.8, 26.4)	1
Rural	137 (10,385)	80.7 (73.6, 86.2)	1.96 (1.04, 3.69)^*^
Economic quintile			
1 (poorest)	138 (9793)	76.1 (66.9, 83.3)	1
2	26 (2046)	15.9 (10.5, 23.4)	0.55 (0.31, 0.98)^*^
3	9 (880)	6.8 (2.7, 16.4)	0.43 (0.14, 1.32)
4	1 (30)	0.2 (0, 1.7)	0.03 (0, 0.28)^**^
5 (richest)	1 (125)	1 (0.1, 6.8)	0.73 (0.08, 6.48)
Home in extreme poverty			
No	87 (6049)	47 (37.5, 56.7)	1
Yes	88 (6825)	53 (43.3, 62.5)	1.83 (1.18, 2.83)^**^
Human Development Voucher			
No	73 (4992)	38.8 (30.5, 47.8)	1
Yes	102 (7882)	61.2 (52.2, 69.5)	2.57 (1.65, 3.98)^b^

^*^Statistically significant difference, *p* value < 0.05. ^**^ Statistically significant difference, *p* value < 0.01

Regarding the characteristics of the mother, having 4–7 children and more than seven children per mother significantly increased the probability of child stunting by 1.94 (95% CI 1.13, 3.24) and 3.24 times (95% CI 1.7, 6.15) respectively, compared to having 1–3 children per mother. Children from economic quintile 2 and 4 were significantly less likely to be stunted, compared to children in the poorest quintile 1 (OR 0.55; 95% CI 0.31, 0.98; OR 0.03; 95% CI 0.00, 0.28, respectively). Children who resided in rural areas, belonged to a household in extreme poverty or received the Human Development Voucher were more likely to be stunted, compared to those who resided in urban areas, did not have extreme poverty or did not receive such a Voucher (OR 1.96; 95% CI 1.04–3.69; OR 1.83; 95% CI 1.18, 2.83; OR 2.57; 95% CI 1.65, 3.98, respectively) (Table 4).

Timely initiation of breastfeeding, exclusive and continued breastfeeding, and receiving food the day before did not show a statistically significant association with stunting on any age group (Table 5). On one hand, the presence of a diverse diet on children from age 19 to 23 months was associated with an OR of 0.22 (95% CI 0.06, 0.86) of presenting stunting, in comparison with those who lacked a diverse diet. However, when an adjusted analysis was performed, this association was lost. On the other hand, children aged 6 to 12 months who did not received the minimum meal frequency, had 3.28 (OR) times (95% CI 1.3, 8.27) the probability of presenting stunting compared to those who did; this association remained significant after adjustment. Finally, regarding the consumption of food rich in iron for children ranging from 13 to 18 months, paradoxically, the odds of being stunted were 3.75 (95% CI 1.25, 11.29) higher for a child that received such nourishment. However, children who received food rich in iron ranging from 19 to 23 months of age, were less likely to be stunted compared to those who did not receive it (OR 0.04; 95% CI 0.00, 0.51). Both were statistically significant.

**Table 5:** Association between stunting (length-for-age Z-score <2 SD) and indicators of lactation and complementary feeding in indigenous children under 2 years of age. National Health and Nutrition Survey, Ecuador, 2012 (n = 625; expanded = 48069)

**Indicator**	**Non-adjusted analysis** **OR (IC95%)**	**Value p**	**Adjusted Analysis**^**a**^ **OR (IC95%)**	**Value p**
Early Breastfeeding				
No	1.00	0.46	1.00	0.91
Yes	0.82 (0.48 – 1.39)		0.96 (0.55 – 1. 69)	
Exclusive breastfeeding in children under 6 months				
No	1.00		1.00	
Yes	0.74 (0.16 – 3.41)	0.39	0.40 (0.05 – 3.42)	0.39
Continuous breastfeeding (7 to 23 months)				
Yes	1.00		1.00	
No	2.81 (1.18 – 6.66)	0.02*	2.91 (1.17 – 7.45)	0.001*
Received food the day before (6-23 months old)				
No	1.00		1.00	
Yes	0.22 (0.08 – 0.62)	0.005*	0.20 (0.08 – 0.60)	0.005*
Food diversity				
No	1		1.00	
Yes	0.91 (0.55 – 1.50)	0.71	0.99 (0.61 – 1.63)	0.99
Minimum meal frequency for age				
Yes	1.00		1.00	
No	2.06 (0.88 – 4.83)	0.93	2.41 (0.95 – 6.15)	0.07
Consumption of food rich in iron				
No	1.00		1.00	
Yes	0.69 (0.42 – 1.15)	0.15	0.69 (0.41 – 1.19)	0.19

^a^OR adjusted for child´s sex, area of residence, number of children, economic quintile and mother’s height

*Value p <0,05

## Discussion

In Latin America, a decrease in child malnutrition has been shown in the last decade. However, children belonging to the indigenous population group are more affected [3]. In Ecuador, according to ENSANUT 2012 data, the prevalence of stunting in children under five is 25.3%, but this average does not show the differences and associated factors in the most affected population, which is indigenous children under five years of age [7].

In this study, the prevalence rate of stunting is very high in children of 12–23 months, reaching a value of 44.6%. Several studies have shown that stunting increases with age. In the National Family Health Survey of India 2005–2006, stunting rates increase to 58% in children aged 18 to 23 months [13]. In local studies, chronic malnutrition was found to be higher in children aged 12 to 23.9 months, compared to children aged 0 to 12 months [14, 15].

In the total population sampled for this study, the predominant residential area was the rural sector, and the highest percentage of those surveyed belonged to the poorest quintile. Mothers had mainly attained a primary or high school education level. Indigenous groups are among the most marginalized segments at a socioeconomic level and are exposed to inadequate sanitation conditions [8]. This combination of factors leads to a high prevalence of stunting in this ethnic population. Several studies involving indigenous people of South America have noted a relationship between deficient environmental conditions and practices related to breastfeeding and the introduction of complementary foods that contribute to chronic malnutrition [8].

In this study, 70% and more of children in the age groups 0–12 months and 12–23 months received timely breastfeeding within the first hour of birth. Seventy-eight-point two percent of children younger than six months were exclusively breastfed, and 94% of children continued to be breastfed over six months of age. Other researchers have shown that breastfeeding practices are observed in a high percentage of indigenous populations worldwide. In Guatemala, 76% of indigenous Mayan women practice timely initiation of breastfeeding [16]. In Brazil, 94.6% of indigenous Xakriabá children were breastfed as their first food [17]. In Canada, 72.5% of indigenous children received timely breastfeeding in the first hour after birth, and 57.9% were exclusively breastfed up to six months [18]. However, it has been observed that the onset of breastfeeding and the duration of breastfeeding have been decreasing in younger indigenous populations as compared to their predecessors, with a higher consumption of Infant milk formulas instead [19], especially in poor populations [20].

More than 70% of the children in the different groups analyzed, received food the day before. However, only the 32.5% of children of 6 to 12 months had a diet with dietary diversity. Studies in indigenous populations suggest that the introduction of food is later in life and with limited diversity [15, 21, 22]. In this study, children who had diversity in their diet were mostly from the rural sector, and were from the richest quintiles. Only 58% and 67.8% of children between 13 to 18 months and 19 to 23 months, respectively, received the minimum number of meals per day for their age. Children who received the minimum meal frequency for their age were primarily from the wealthiest quintiles. Other studies also found better indicators of complementary feeding in populations with better socioeconomic levels [15, 20].

In this study, indicators such as EBF or timely initiation of breastfeeding were not associated with stunting on any age group, result that coincides with findings of other authors [23, 24]. Other studies have found that any breastfeeding practice is a protective factor against stunting [25–27]. However, in relation to infants who have been exclusively breastfed, several studies did not find a significant impact on growth compared to children who were not exclusively breastfed [23, 28]. Several studies have found mixed results for the relationship between breastfeeding and feeding practices with stunting. Some studies have higher rates of mild and severe malnutrition in breastfed children [29–31]. At first glance, it may seem that better breastfeeding practices could be related to higher rates of stunting or worse child nutritional status, however; this may be explained by a reverse causality. For instance, a mother of a child that already has a poor nutritional status, or several health problems, such as diarrhea, or who lives in a community with high prevalence of malnutrition, could try to improve the child’s health by having better breastfeeding practices, thus, the association between stunting and breastfeeding [32]. It has also been found that in contexts of food insecurity with high prevalence of breastfeeding, breastfeeding allows the accumulation of body fat, which would contribute to maintaining the child's weight and height [24]. Either way, there is enough evidence to show the breastfeeding has additional benefits, such as protection from infectious diseases and others related to a child’s survival [10]. Breastfeeding plays a role in the immune system that protects the infant from infections, including diarrheal diseases, in addition to avoiding exposure to unsafe liquids or foods such as unsafe water [29, 30].

The socioeconomic characteristics, the lack of minimum frequency of meals and not consumption of food rich in iron were predominant in the association with stunting [25, 26, 33, 34]. Not achieving the minimum meal frequency for age was significantly associated with stunting in children from age 6 to 12 months. This data agrees with other publications that provide evidence for reaching the minimum meal frequency for age as a protective element for linear growth [33]. Several studies have shown that children between 6 and 8 months of age do not meet the adequate energy intake requirements, which worsens their nutritional status; this inadequate intake extends to children between 9 and 11.9 months of age [15]. As explained previously, the first two years of a child’s life are essential for their adequate growth and development, and yet that is the same period during which the prevalence of stunting has been shown to be highest. Therefore, there is sufficient evidence to suggest that adequate complementary feeding is essential to reduce the incidence of stunting in children [1, 23, 24, 26, 28]. Additionally, and paradoxically, children from 13 to 18 months of age that have eaten foods rich in iron was related with a higher probability of stunting, however, this may be explained by the reverse causality previously explained [32].

Indigenous populations, especially those that live in the inter-Andean valleys like those in Ecuador, live at high altitudes in isolated communities where children are exposed to deficiency of macro and micronutrients due to scarcity, which, in turn, affects their linear growth [35–38]. It is important to consider that complementary feeding may be introduced with foods that have a lower energy and nutrient content, or disease-transmitting microorganisms may be introduced by inadequate sanitation conditions [8]. These elements could further condition the growth possibilities of indigenous children. Therefore, it is necessary to strengthen the safe and healthy introduction of food in children, which must go hand by hand with food security programs for indigenous populations.

There are some limitations in this study. As it is a study based on secondary data, there are some elements related to stunting that could not be evaluated, such as variables referring to recurrent morbidity, birthweight, daily calorie intake, and cultural elements related to diet. As it is a cross-sectional study, the results of the associations obtained do not show causality with stunting. Since a temporary cause-effect relationship cannot be evidenced, we cannot strictly speak of risk or prevention factors. Data collection could lead to memory biases linked to mothers' responses. Likewise, the mother could have given positive responses in breastfeeding or feeding practices to satisfy the researcher. Additionally, the analysis of nutritional indicators is based solely on the mother's information about the 24 h prior to being surveyed, which does not allow for a more in-depth analysis of the food consumption situation over time or the specific number of calories consumed. Nor was it possible to discriminate the results of the breastfeeding indicators among breastfed children who receive a bottle at the same time, due to missing data in the responses given by mothers on bottle feeding and infant milk formula consumption, which led to a high variability in the results.

However, the study presents important evidence on the prevalence of breastfeeding and feeding practices in vulnerable indigenous populations in Ecuador, which is characterized by a high prevalence of stunting. Likewise, it explores factors that limit compliance with breastfeeding and complementary feeding practices. On the other hand, this study reveals novel information that would associate the lack of a minimum number of meals per day and a diet low in iron with a higher probability of stunting in indigenous children under two years of age. Additionally, this study shows the relevance of an adequate transition of breastfeeding and feeding practices after 6 months of age, which would be associated with greater stunting in the indigenous population under two years of age.

## Conclusions

There is a high prevalence of stunting in indigenous children in Ecuador, that affects 44.6% of children between 12 and 23 months of age. The traditionally excluded indigenous population has a history of malnutrition, which adds to their social vulnerability, which would affect their development and future life. Policies and health plans for the prevention of stunting are required to consider the multi-causes of this health problem, including the promotion of breastfeeding and complementary feeding.

Although the prevalence of breastfeeding is high in the indigenous population, there are gaps in complementary feeding indicators from six months of age, which are related to socioeconomic factors. Thus, the poorest or less educated households present less frequent and diversity of diet in their children under two years of age.

Not meeting the minimum meal requirement for age for children from 6 to 12 months of age was associated with stunting in indigenous population. On the other hand, receiving food rich in iron for children from age 19 to 23 months was associated with a lower probability of stunting. The association found here with complementary feeding demonstrates the importance of feeding practices in the growth of indigenous children, in a context of vulnerability. Interventions focused on the underlying causes of child stunting are required to reduce the vulnerability of this population group.

For indigenous populations, improving socioeconomic conditions, including the education of mothers, is a long-term initiative. It is also necessary to improve the hygienic and sanitary conditions of the population, promote breastfeeding and food security, generate adequate access to local foods that favors the consumption of foods rich in protein and local cultural eating practices, to reduce the high prevalence of stunting in children under two years of age. National plans and strategies are necessary to promote continuous breastfeeding and adequate introduction of food in the indigenous population.

## Supplementary Information

**Additional file 1.** Distribution of children included in the study by province and city of residence.

## Data Availability

The data that supports the findings of this study is openly available in: Instituto. Nacional de Estadísticas y Censos (INEC). (2012) Encuesta Nacional de Salud, Salud. Reproductiva y Nutrición (ENSANUT)-2012, at https://www.ecuadorencifras.gob.ec/encuesta-nacional-de-salud-salud-reproductiva-y-nutricion-ensanut-2012/, reference [11]

## Abbreviations

CI: Confidence interval
EBF: Exclusive breastfeeding before 6 months
ENSANUT: National Health and Nutrition Survey of Ecuador
INEC: National Institute of Statistics and Censuses
OR: Odds Ratio
WHO: World Health Organization
