# Supplementary material for: Breastfeeding, feeding practices and stunting in indigenous Ecuadorians under 2 years of age
Source: Int Breastfeed J. 2022 Mar 5;17:19. doi: 10.1186/s13006-022-00461-0 (PMC8898506; doi:10.1186/s13006-022-00461-0)

Additional file 1. Distribution of children included in the study by province and city of residence.


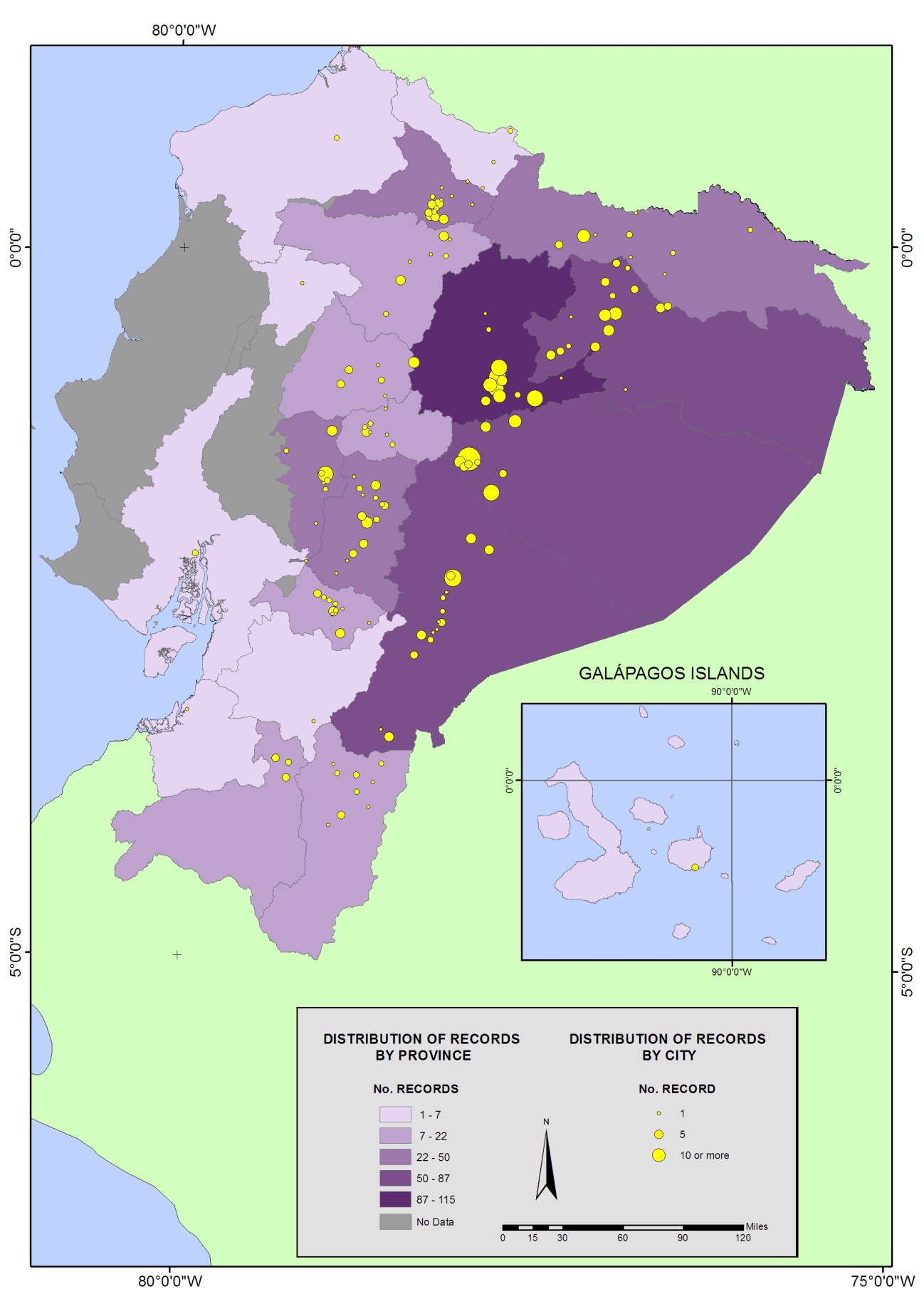

Supplement: Supplementary file 1 — Additional file 1. Distribution of children included in the study by province and city of residence. [file 13006_2022_461_MOESM1_ESM.docx]
